# Enterovirus A71 Meningoencephalitis Outbreak, Rostov-on-Don, Russia, 2013

**DOI:** 10.3201/eid2108.141084

**Published:** 2015-08

**Authors:** Ludmila V. Akhmadishina, Marina V. Govorukhina, Evgeniy V. Kovalev, Svetlana A. Nenadskaya, Olga E. Ivanova, Alexander N. Lukashev

**Affiliations:** Chumakov Institute of Poliomyelitis and Viral Encephalitides, Moscow, Russia (L.V. Akhmadishina, O.E. Ivanova, A.N. Lukashev);; Center of Hygiene and Epidemiology of Rostov Region, Rostov-on-Don, Russia (M.V. Govorukhina);; Administration of Russian Federal Consumer Rights Protection and Human Health Control Service of Rostov Region, Rostov-on-Don (E.V. Kovalev, S.A. Nenadskaya)

**Keywords:** enterovirus 71, EV-A71, human enterovirus, meningitis, meningoencephalitis, neuroinfection, outbreak, Russia, viruses

## Abstract

Seventy-eight cases of enterovirus infection, including 25 neuroinfections, occurred in Rostov-on-Don, Russia, during May–June 2013. The outbreak was caused by an enterovirus A type 71 (EV-A71) subgenotype C4 lineage that spread to neighboring countries from China ≈3 years earlier. Enterovirus associated neuroinfection may emerge in areas with a preceding background circulation of EV-A71 with apparently asymptomatic infection.

Enterovirus A type 71 (EV-A71; family *Picornaviridae*, genus *Enterovirus*, species *Enterovirus A*) is the most neuropathogenic nonpolio enterovirus in humans. Over the past 30 years, this virus has caused many outbreaks and epidemics of hand, foot and mouth disease (HFMD) with neurologic complications in the Asia-Pacific region and China (*1*). In Europe, EV-A71 commonly causes asymptomatic infection (*2*–*4*) and is only occasionally associated with severe neuroinfection (*3*,*5*). The last epidemics of EV-A71 infection in Europe occurred during 1975–1978 (*6*,*7*).

## The Study

**Figure 1:**
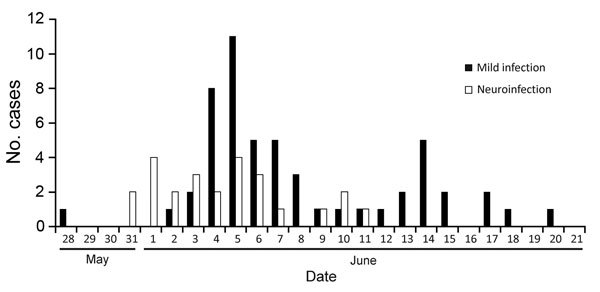
Incidence of neuroinfection during an enterovirus A type 71infection outbreak among children attending a childcare facility, Rostov-on-Don, Russia, 2013. Of 78 infected children (1–7 years of age), 25 experienced neuroinfection (meningitis or meningoencephalitis) and 53 experienced mild infection (hand, foot and mouth disease or fever).

During 2013, an outbreak of EV-A71 infection occurred in Rostov-on-Don, a city in southern Russia. The first case was diagnosed on May 31 in a 2-year-old boy. A cerebrospinal fluid sample from the boy contained EV-A71 and pneumococcal bacteria, suggesting combined bacterial and viral meningoencephalitis. The boy died of meningoencephalitis on day 5 after disease onset. According to the official State Surveillance System report, 366 children (1–7 years of age) attended the same childcare facility as the boy who died, and over the 3 weeks after the index case, 77 more children were involved in the outbreak (Figure 1). Of the 78 children, 43 were boys and 35 were girls. Fourteen children were 1–2 years of age, 63 were 3–6 years of age, and 1 was 7 years of age. A total of 68 children were hospitalized, of whom 25 (15 boys, 10 girls) had meningitis or meningoencephalitis; 6 of these children were 1–2 years of age, and 19 were 3–6 years of age.

Enterovirus RNA was detected in fecal samples (n = 53), throat swab samples (n = 23), or both (n = 17) from 59 of the 78 patients. EV-A71 was identified by partial viral protein 1 genome region sequencing in the cerebrospinal fluid sample from the first patient and in fecal and throat swab samples from the other patients. The outbreak was officially defined as including only those children from the childcare facility attended by the index patient. Additional cases of HFMD and meningitis that occurred outside that kindergarten were not officially accounted for in outbreak statistics; thus, it is likely that the outbreak size was underestimated. Simovanian et al. (*8*) indicated that a total of 139 EV-A71 infections were reported in Rostov-on-Don during May–June 2013. According to that report, 30.2% of patients had meningitis and 7.2% had meningoencephalitis. Of note, exanthema was observed in only 79.9% of patients.

The ratio of neuroinfection (meningitis and meningoencephalitis) cases relative to mild infection (i.e., HFMD and fever) cases was unusually high in this outbreak. Among the 78 officially reported cases, 25 (32.1%) were meningitis or meningoencephalitis. After the first fatal case, all children in the childcare facility were proactively screened for enterovirus infection symptoms, so it is unlikely that many patients with mild infection were missed. Asymptomatic children were not screened for EV-A71 RNA. However, the ratio of meningitis cases was high even relative to the total number of children attending the childcare facility (6.8% [25/366 children]). This finding differs from those from a 1998 outbreak in Taiwan, in which 405 (0.3%) of 129,106 persons with HFMD had severe disease (i.e., fever >38°C or neurologic manifestations) (*9*). Thus, the proportion of severe disease in a localized setting can be substantially higher than that for an epidemic overall.

Thirteen isolates from the EV-A71–infected children were available for study. Viruses were propagated in cell culture, and the entire viral protein 1 genome region (891 nt) was amplified as described previously (*10*). The outbreak samples differed from each other by <3 nt substitutions (0.3%). Furthermore, they shared 97%–98% nt sequence identity with several subgenotype C4 viruses isolated in China during 2009–2011, indicating that the strains belonged to this subgenotype.

**Figure 2:**
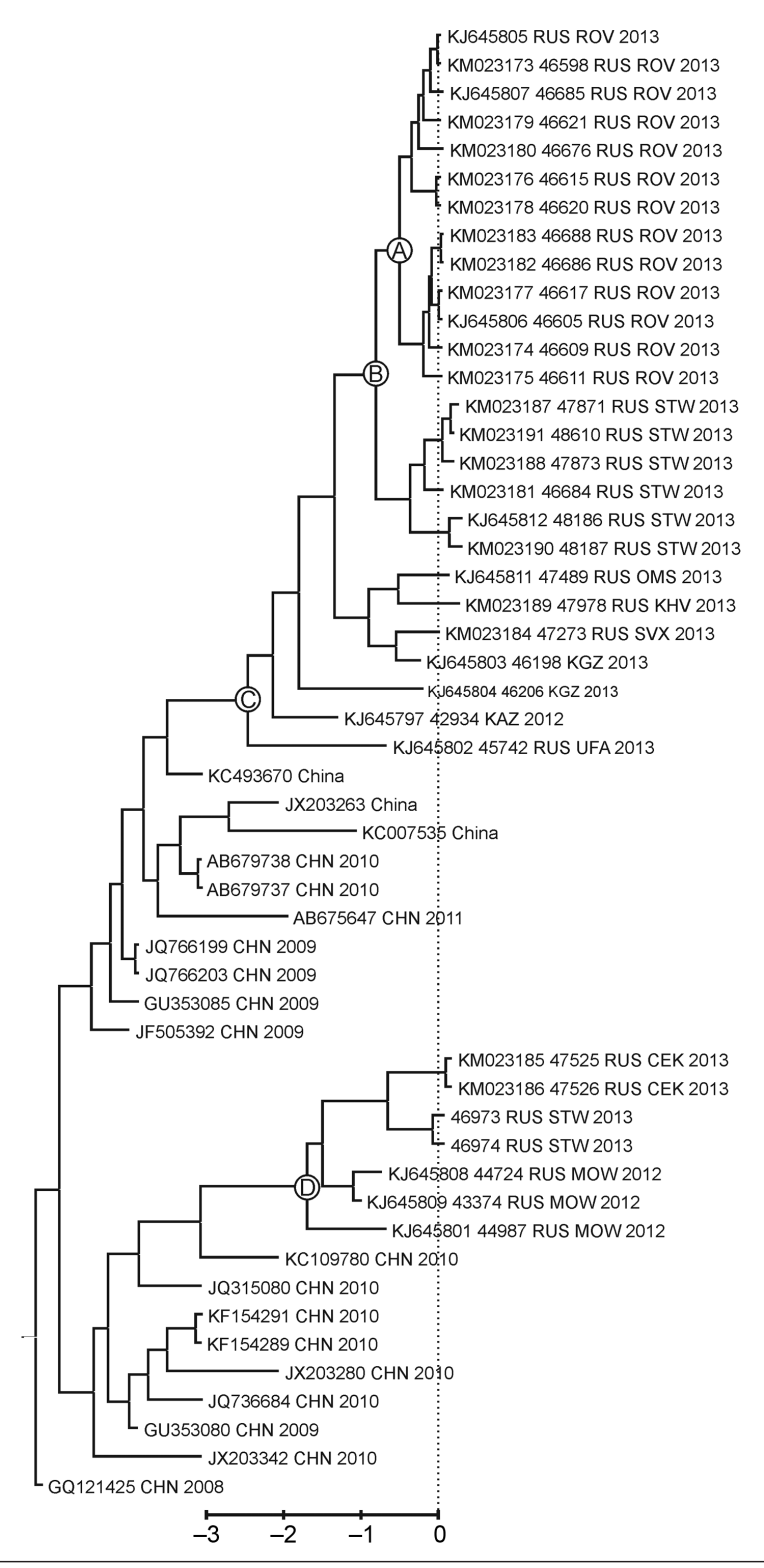
Phylogenetic tree comparing sequences of outbreak and other enterovirus A type 71 (EV-A71) subgenogroup C4 strains isolated in Russia during 2012–2013 with the most closely related sequences in GenBank. Complete viral protein 1 genome regions (891 nt) were compared. The tree was reconstructed by using a coalescent Bayesian algorithm implemented in BEAST 1.7.5 (*11*) with the SRD06 substitution model, relaxed exponential clock, and constant population prior. The dataset included 63 GenBank sequences that were most similar to the outbreak virus, as determined by using BLAST (http://www.ncbi.nlm.nih.gov/blast/Blast.cgi), and 22 EV-A71 subgenogroup C4 sequences obtained elsewhere in Russia, Kazakhstan, and Kyrgyzstan during 2011–2013 (*10*). Only the part of the tree that is relevant to the discussion is shown. Strain names contain the GenBank or internal reference number, country of isolation, city code for Russian isolates, and year of isolation. City codes: CEK, Chelyabinsk; CHN, China; KAZ, Kazakhstan; KGZ, Kyrgyzstan; KHV, Khabarovsk; MOW, Moscow; OMS, Omsk; ROV, Rostov; RUS, Russia; STW, Stavropol; SVX, Yekaterinburg; UFA, Ufa. GenBank accession numbers for previously unpublished viruses (KM023173–KM023191‏) are shown in the tree. The dotted line shows the outbreak onset on May 31 (some strains were isolated after that date). Circled letters indicate sequences for viruses isolated in Rostov-on-Don and the Rostov Region of Russia (A); Stavropol region of Russia, which neighbors Rostov Region (B); Siberia, the Pacific region of Russia, Kazakhstan, and Kyrgyzstan (C); and Stavropol, central Russia and Ural Region (D). Scale bar indicates year before the 2013 outbreak.

Phylogenetic analysis of the virus spread was performed by using a Bayesian phylogenetic approach implemented in BEAST version 1.7.5 (*11*). The substitution rate in this dataset was 6.8 × 10^−3^ substitutions per site per year, which is similar to rates reported for EV-A71 subgenogroup C4 in other studies (*12*,*13*). Sequences from Rostov-on-Don and the Rostov region were monophyletic (Figure 2, node A); the time to the most recent common ancestor was 200 days (95% high probability density interval 68–347 days); however, this group was imperfectly supported by a posterior probability value of 0.85. The high number of days to the most recent common ancestor of the outbreak strain is surprising because the outbreak presented as a point-source expansion with a traceable source and transmission chains. A possible explanation for this finding might be that that the substitution rate of EV-A71 might accelerate substantially during an outbreak, or additional substitutions might have been introduced upon isolation in cell culture.

In the phylogenetic tree, isolates from Rostov-on-Don grouped (with a posterior probability of 1) with an additional 6 viruses that were isolated in the neighboring Stavropol region during and up to 2 months after the outbreak (Figure 2, node B); however, no cases of neuroinfection were reported in that region. Isolates from Rostov-on-Don and Stavropol grouped reliably with another 7 viruses isolated in Siberia, the Pacific region of Russia, Kazakhstan, and Kyrgyzstan during 2012–2013 (Figure 2, node C). The time to the most recent common ancestor of these viruses was 921 days (95% high probability density range 665–1,195 days) before the outbreak. Therefore, the virus variant that caused the outbreak was likely introduced to Russia or Middle Asia ≈3 years before the outbreak and circulated extensively without causing notable illness. Of interest, another lineage of EV-A71 subgenogroup C4 representing an independent introduction from China was circulating at the same time in Central Russia and Stavropol region (Figure 2, node D).

## Conclusions

Over the past 2 decades, EV-A71 became a prominent emerging virus in Asia. However, the EV-A71 epidemiologic situation remained calm in Russia, Europe, and North America, despite common circulation of the virus, as suggested by surveillance and seroepidemiologic studies (*3*,*4*). According to our previous study (*10*), EV-A71 was circulating in Rostov-on-Don before 2008, but EV-A71 seroprevalence among infants 1–2 years of age (4.5%) was substantially lower than that observed in other regions of Russia (9.9%–22.7%). It is possible that lower herd immunity could have facilitated the outbreak we describe here. Our findings show that an outbreak of EV-A71 neuroinfection may emerge in new areas despite preceding background circulation of EV-A71 with apparently asymptomatic infection.
